# Manufacturing micropatterned collagen scaffolds with chemical-crosslinking for development of biomimetic tissue-engineered oral mucosa

**DOI:** 10.1038/s41598-020-79114-3

**Published:** 2020-12-17

**Authors:** Ayako Suzuki, Yoshihiro Kodama, Keito Miwa, Kazuma Kishimoto, Emi Hoshikawa, Kenta Haga, Taisuke Sato, Jun Mizuno, Kenji Izumi

**Affiliations:** 1 Division of Biomimetics, Faculty of Dentistry and Graduate School of Medical and Dental Sciences, Niigata University, Niigata, Japan; 2 Division of Pediatric Dentistry, Faculty of Dentistry and Graduate School of Medical and Dental Sciences, Niigata, Japan; 3 Taki Chemical Co., Ltd, Kakogawa, Japan; 4 Department of Electronic and Physical Systems, Waseda University, Shinjuku, Tokyo, Japan; 5 Center for Transdisciplinary Research, Institute for Research Promotion, Niigata, Japan; 6 Research Organization for Nano and Life Innovation, Waseda University, Shinjuku, Tokyo, Japan

**Keywords:** Biomaterials, Soft materials, Biomaterials, Biotechnology, Tissue engineering

## Abstract

The junction between the epithelium and the underlying connective tissue undulates, constituting of rete ridges, which lack currently available soft tissue constructs. In this study, using a micro electro mechanical systems process and soft lithography, fifteen negative molds, with different dimensions and aspect ratios in grid- and pillar-type configurations, were designed and fabricated to create three-dimensional micropatterns and replicated onto fish-scale type I collagen scaffolds treated with chemical crosslinking. Image analyses showed the micropatterns were well-transferred onto the scaffold surfaces, showing the versatility of our manufacturing system. With the help of rheological test, the collagen scaffold manufactured in this study was confirmed to be an ideal gel and have visco-elastic features. As compared with our previous study, its mechanical and handling properties were improved by chemical cross-linking, which is beneficial for grafting and suturing into the complex structures of oral cavity. Histologic evaluation of a tissue-engineered oral mucosa showed the topographical microstructures of grid-type were well-preserved, rather than pillar-type, a well-stratified epithelial layer was regenerated on all scaffolds and the epithelial rete ridge-like structure was developed. As this three-dimensional microstructure is valuable for maintaining epithelial integrity, our micropatterned collagen scaffolds can be used not only intraorally but extraorally as a graft material for human use.

## Introduction

Biomaterials are made of natural and synthetic substrates. Recent advances in tissue engineering and regenerative medicine have used biomaterials to develop a variety of constructs of human tissues/organs and to repair or regenerate damaged and diseased human tissues. Three-dimensional (3D) design is one of the ultimate goals of regenerative engineering, which can provide a unique microenvironment for mechanical support and biological signals, and modulating cell behaviors. Therefore, the composition and microstructure of biomaterials, such as their internal structure and surface topography mimicking in vivo tissues, are recent interests. For example, enhanced patency has been shown using a poly-vinyl alcohol vascular graft with a micropatterned luminal surface (grating), which endothelialized after in vivo implantation, when compared with nonpatterned grafts^1^.

Rete ridges are undulating/interdigitating microstructures found between the epithelial cell layer and the underlying connective tissue in human epithelial tissues^2,3^. This intrinsic and characteristic topographical feature, called the dermal–epidermal junction (DEJ) in skin, enhances the adhesion of those two layers due to an increase in surface area of the DEJ^4^. This microstructure allows the avascular epidermal layer access to the nutrient supply from capillary networks in the dermis, providing vital functions for skin homeostasis. Since the DEJ is a niche for epidermal stem cells, this inherent 3D microarchitecture provides a significant microenvironment for other epithelial tissues, such as the oral mucosa, small intestinal mucosa, gastric mucosa and corneal limbus^5–8^. Nonetheless, the characteristic topography of the DEJ-like structure is markedly different among various epithelial tissues^5–8^. Furthermore, despite its significant roles, rete ridges have not been engineered into epithelial tissue constructs used in clinical applications, resulting in a linear, not 3D interface between epithelial layers and scaffolds^9^.

Micropatterning of scaffolds is another potentially effective strategy because it recreates the biomimetic microenvironment and assists in understanding the fundamental mechanisms underlying a keratinocyte stem cell niche. To address this challenge, many nano- and micro-fabrication techniques to mimic and reproduce the characteristic microstructure have been utilized^10^. Although micro/biofabrication technologies to engineer scaffolds have emerged, in particular natural hydrogel scaffolds, there have been no studies on a tissue-engineered oral mucosa constructs with the geometric features at the interface between cells and scaffold^10–13^.

We previously reported the development of microstructured collagen scaffolds, derived from fish-scales, to manufacture a tissue-engineered oral mucosa equivalent (EVPOME). Our technique, which used a micro electro mechanical systems (MEMS) process and soft lithography for fabricating negative mold, enabled the creation of a connective tissue papillae-like micropattern on a 1% collagen scaffold that mimicked oral mucosa in vivo. The original design of four micropattern prototypes was well-preserved and transferred onto the scaffolds. However, histological examinations of the EVPOME showed flattening of the vertical dimension and expansion of pitches in the microstructures, resulting in loss of biomimetic cues and failure of the aims. Therefore, the properties of the micropatterned portion need to be improved.

Reconstituted collagen is stable in aqueous media, but the collagen hydrogel typically lacks the physical properties of native tissues, partially due to incomplete molecular crosslinking^14^. Thus, the crosslinking process can be harnessed to modulate the properties of collagen matrices. A water-soluble crosslinker, 1-ethyl-3-(3-dimethylaminopropyl) carbodiimide (EDC), has been widely used to reinforce substrates, such as collagen-based biomaterial for tissue reconstruction, suggesting many benefits. In the present study, we designed and fabricated negative molds with four micropattern prototypes, with smaller dimensions and different aspect ratios, using soft lithography, and then tested the versatility of our manufacturing system. Next, we examined the effect of EDC as a chemical crosslinker, instead of the γ-irradiation previously used to crosslink, on the physical property of collagen scaffold to enhance the mechanical characteristics. Finally, we investigated the histologic findings of a tissue-engineered oral mucosa equivalent to evaluate the effects of rete ridge-like microarchitectures of the collagen scaffolds on epithelial tissue regeneration.

## Results

### Fabrication of negative molds for micropatterns with various dimensions and aspect ratios

**Figure 1:**
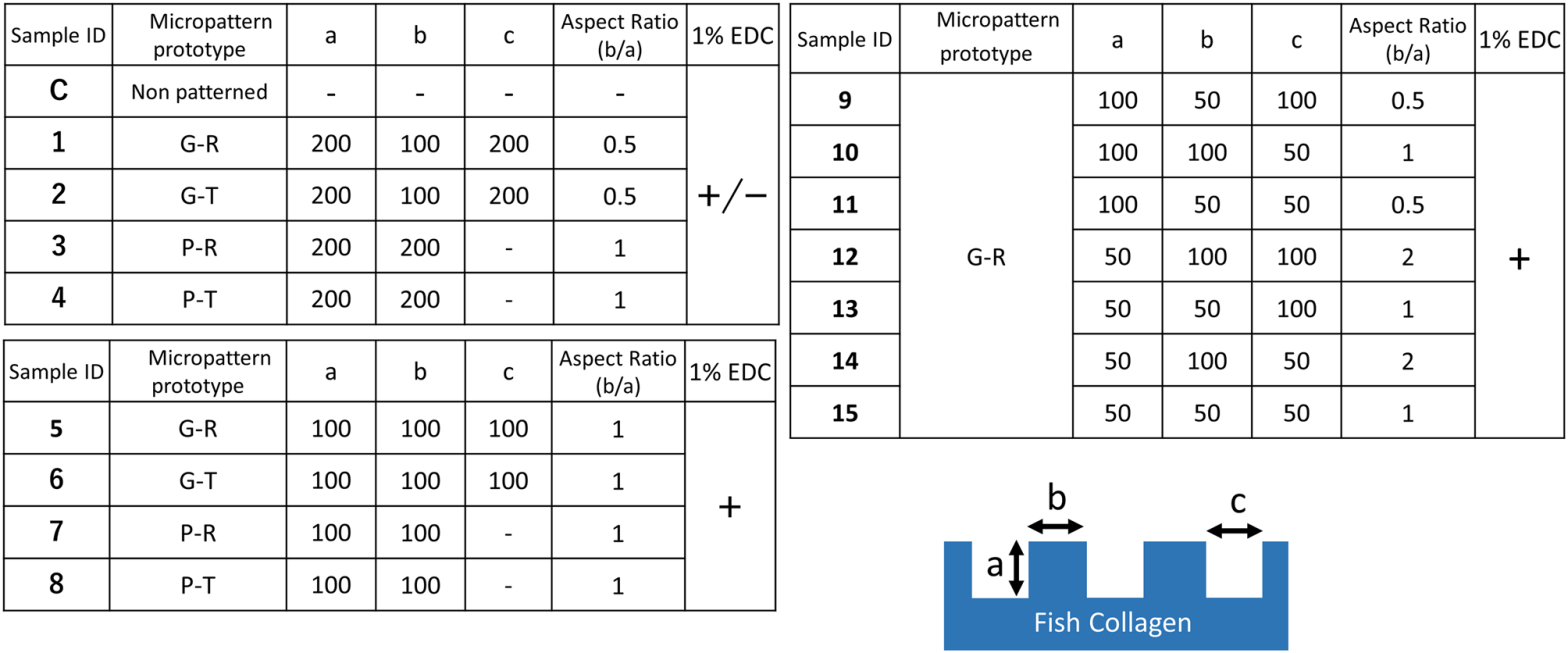
A total of 16 micropatterns of collagen scaffolds designed in this study (C, 1–15). The scaffolds include several combinations of four prototypes, with different feature dimensions and aspect ratios, as well as presence and absence of EDC crosslinking (1–4). G–R, G–T, P–R, and P–T represent for grid pattern with rectangular configuration, grid pattern with truncated configuration, pillar pattern with rectangular configuration, and pillar patterns with truncated configuration, respectively. Schematic illustration at the bottom of right side shows a basic micropattern design referred in the table within this figure [(**a**) height, (**b**) thickness, (**c**) channel width].

**Figure 2:**
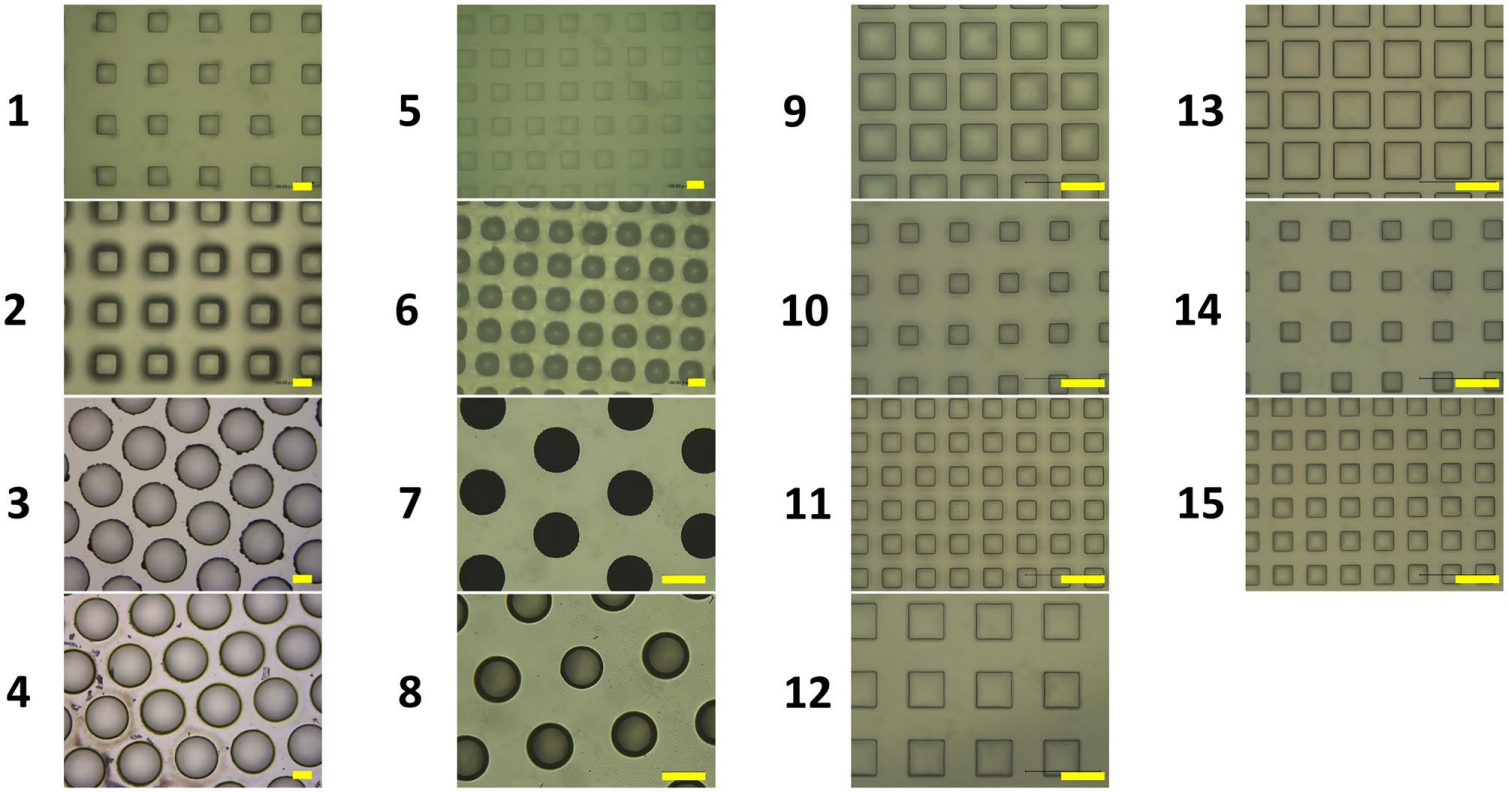
Optical microscopy images of the top view of negative molds made of PDMS (1, 2, 5, 6, 9–15) and Si (7, 8) substrates (Magnification: 1, 2, 5, 6 200 × ,7–15 400 ×). Confocal microscopy images of the top view of negative molds made of Si (3,4) substrates (Magnification: 3, 4 200 ×) (Scale bar = 100 μm).

We previously developed negative molds for microstructure formation on a collagen scaffold using polydimethylsiloxane (PDMS) and Si substrates for grid- and pillar-micropattern prototypes, with both rectangular and truncated configurations, respectively. This molding system used MEMS process and soft lithography. This enabled us to fabricate the negative molds needed in this study to create micropatterned collagen scaffolds with different feature dimensions, which included height, thickness, and channel width (Figs. 1 and 2). Measurement of the 15 micropatterns showed that the variations were almost within 25% of the originally designed dimensions (Table 1). Similar to our previous study, variation of more than 10% was observed in the dimensions of negative molds for only grid patterns (Sample ID 1–4). For other micropatterns, variation of more than 10% was observed in the truncated prototypes of the sample group of 5–8 and in the channel width of the sample group of 9–15.

**Table 1:** Measurements of 15 different negative molds after fabrication as well as their variations as compared with the originally designed dimensions for both PDMS substrates for the grid micropattern formation and Si substrates for pillar micropattern formation (n = 3).

Micropattern prototype	Dimension originally designed [μm]	Measurement (n = 3) [μm]	Variation [%]
**Sample ID 1–4**			
**1**			
*Grid-rectangular*			
Height	200	206.0 ± 0.82	+ 3.0
Thickness	100	117.0 ± 0.47	+ 17.0
**2**			
*Grid-truncated*			
Height	200	227.0 ± 2.90	+ 14.0
Thickness	100	119.0 ± 2.90	+ 19.0
**3**			
*Pillar-rectangular*			
Height	200	206.0 ± 1.30	+ 2.8
Diameter	200	200.0 ± 0.00	0.0
**4**			
*Pillar-truncated*			
Height	200	184.0 ± 5.60	− 7.8
Diameter	200	201.0 ± 0.00	+ 0.5
**Sample ID 5–8**			
**5**			
*Grid-rectangular*			
Height	100	96.3 ± 2.90	− 3.7
Thickness	100	107.0 ± 0.82	+ 7.0
6			
*Grid-truncated*			
Height	100	85.3 ± 1.90	− 14.7
Thickness	100	119.0 ± 2.90	+ 19.0
**7**			
*Pillar-rectangular*			
Height	100	100.0 ± 0.00	0.0
Diameter	100	114.0 ± 0.82	+ 14.0
**8**			
*Pillar-truncated*			
Height	100	75.9 ± 0.48	− 24.1
Diameter	100	80.7 ± 1.90	− 19.3

Micropattern prototype: grid-rectangular	Dimension originally designed [μm]	Measurement (n = 3) [μm]	Variation [%]
**Sample ID 9–15**			
**9**			
Height	100	90.1 ± 2.15	− 9.9
Thickness	50	51.8 ± 0.35	3.6
Channel width	100	83.5 ± 2.14	− 16.5
**10**			
Height	100	94.5 ± 5.31	− 5.5
Thickness	100	100.0 ± 0.76	0.2
Channel width	50	37.5 ± 1.25	− 25.1
**11**			
Height	100	91.3 ± 1.89	− 8.7
Thickness	50	52.0 ± 0.35	4.0
Channel Width	50	38.1 ± 1.20	− 23.8
**12**			
Height	50	48.5 ± 1.10	− 2.9
Thickness	100	95.2 ± 1.25	− 4.8
Channel width	100	86.9 ± 0.69	− 13.1
**13**			
Height	50	46.3 ± 2.52	− 7.4
Thickness	50	51.0 ± 0.60	2.0
Channel Width	100	85.4 ± 0.00	− 14.6
**14**			
Height	50	45.7 ± 0.35	− 8.6
Thickness	100	98.5 ± 1.29	− 1.5
Channel width	50	42.0 ± 1.31	− 16.1
**15**			
Height	50	46.1 ± 0.60	− 7.8
Thickness	50	52.2 ± 1.85	4.5
channel width	50	39.1 ± 0.35	− 21.8

**Figure 3:**
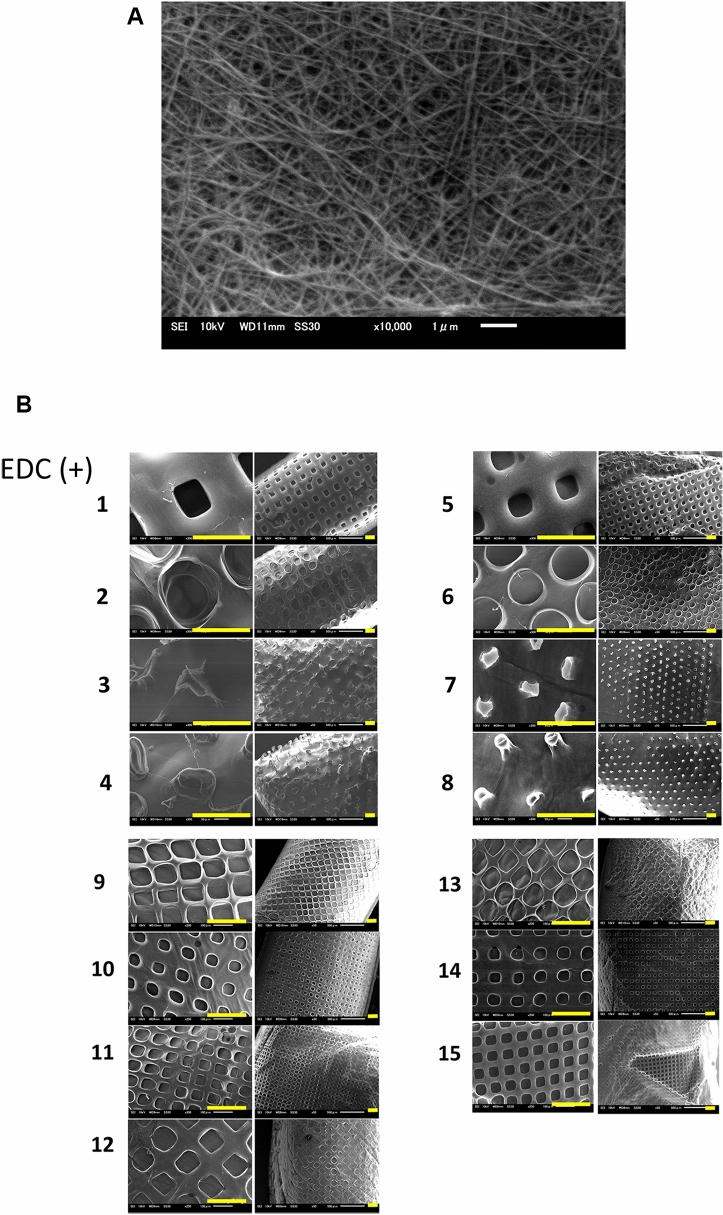
(**A**) Scanning microscopic image of the collagen matrix crosslinked with 1% EDC. The diameter of 1% tilapia scale collagen fibrils after crosslinking was ranged from 40 to 120 μm (n = 5) although it was 30 to 100 μm before crosslinking (compared with the Fig. 3(a) in our previous study^15^. (Magnification　10,000 × ; Scale bar = 1 μm). (**B**) Scanning microscopic images of the collagen scaffolds with 15 different micropatterns’ design shown in Fig. 1. (Magnification: Left panels 1–8 300 × , 9–15 200 × , Right panels 50 ×) (Scale bar = 200 μm).

Scanning electron microscopy (SEM) revealed a well-developed fibril network within the collagen matrices crosslinked with 1% EDC (Fig. 3A). The diameters of fibrils of the 1% tilapia scale type I collagen matrices with EDC cross-linking ranged from 40 to 120 nm, which were approximately 1.2 times thicker than those without EDC crosslinking shown in the Fig. 3a in our previous report^15^. SEM showed that micropatterns were formed on the collagen scaffolds crosslinked with 1% EDC, with several dimensions and aspect ratio. The surface textures of collagen scaffolds with or without 1% EDC crosslinking showed little differences between all the micropatterns^15^. Similar to the previous report, the microstructure configuration of the grid micropattern in this study appeared to be well-preserved, however, the pillar micropattern appeared to be collapsed and poorly preserved, (Fig. 3B; 1–4)^15^. In contrast, the four types of micropatterns that were fabricated with 100 μm in height, thickness and channel width respectively, were all well-transferred onto the collagen scaffold (Fig. 3B; 5–8). Furthermore, for the grid–rectangular (G–R) prototype, the dimension of microstructure configurations smaller than 100 μm was also well-preserved (Fig. 3B; 9–15). Thus, the present study showed that our technique also enabled the fabrication the micropatterns on the collagen scaffolds as small as 50 µm.

### Physical property of collagen scaffold with or without 1% EDC crosslinking

**Figure 4:**
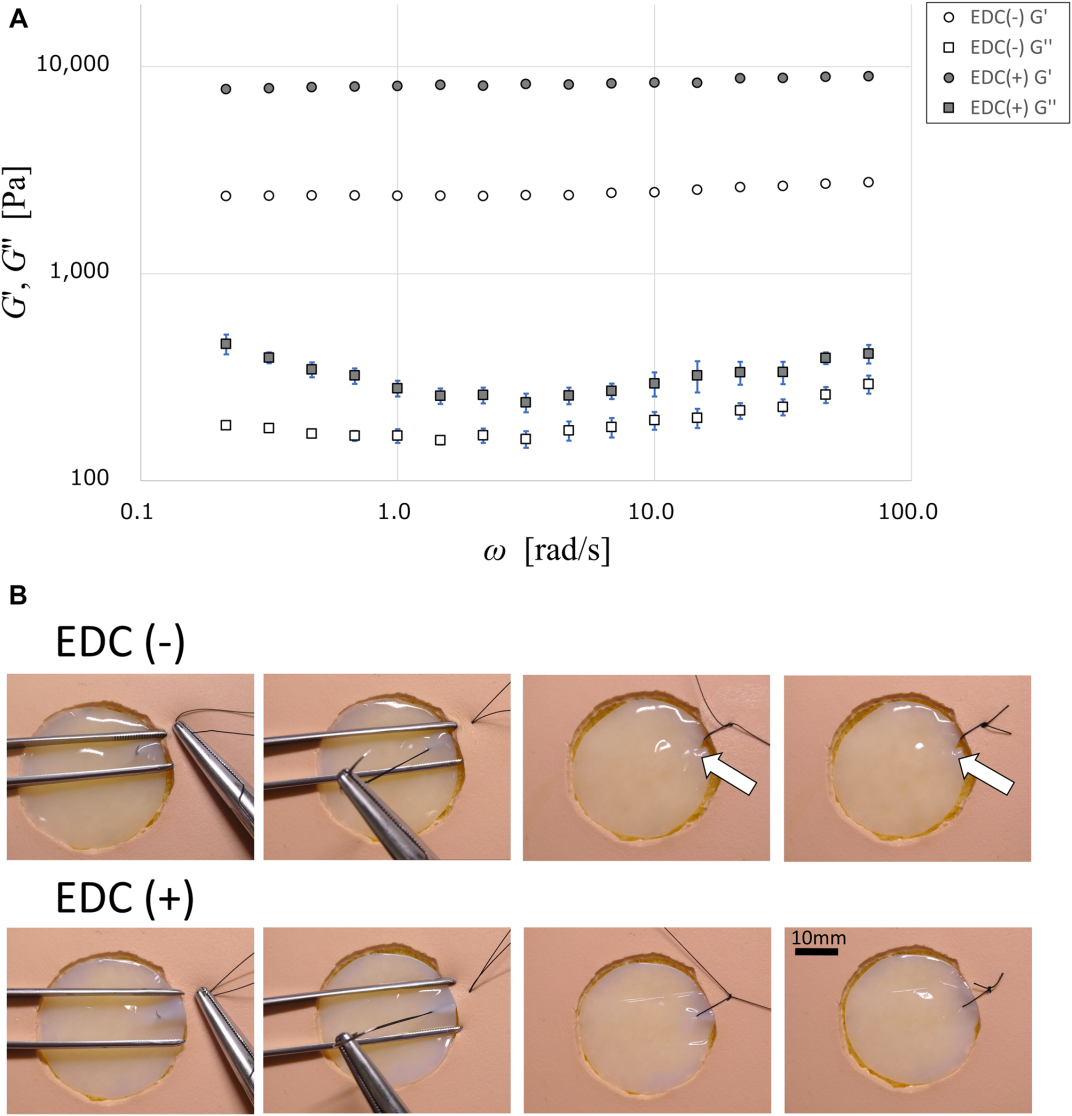
(**A**) Dynamic viscoelastic property of the collagen scaffolds with or without 1% EDC crosslinking measured by a rotational rheometer (n = 5). (**B**) Representative macroscopic image of the collagen scaffold sutured with the epithelial defect of Bio-SKiN using 4–0 braided silk after measurement of dynamic viscoelastic property (n = 5) (Scale bar = 10 mm). All of the collagen gels without crosslinking (top panels) was ruptured when suturing (white arrows).

The collagen scaffold with 1% EDC crosslinking increased the Young’s module compared with the scaffold without crosslinking (approx. 48.65 kPa vs. 29.10 kPa, at 37 °C). This is in contrast to the previous data describing the physical property of the collagen gel mixed with 1.0% chondroitin sulfate (approx. 7.0 kPa). Thus, the physical property of the EDC-crosslinked collagen gels used in this study was enhanced, compared with the previous study. The dynamic viscoelastic property of the collagen scaffold, measured using a rotational rheometer with a parallel plate configuration, is shown in Fig. 4A, where storage modulus *G*′ and loss modulus *G*″ are plotted against frequency *ω*. The results showed *G*′ is higher than *G*″ and takes a plateau value over the whole frequency range measured in this study. Such frequency dependence corresponds to the behavior expected of an elastic gel. Furthermore, when using a 4–0 braided silk suture and a round needle, the collagen scaffold with 1% EDC crosslinking was durable enough to be sutured in contrast to the scaffold without crosslinking, which was readily ruptured (Fig. 4B).

### Macroscopic findings of EVPOMEs during manufacturing

**Figure 5:**
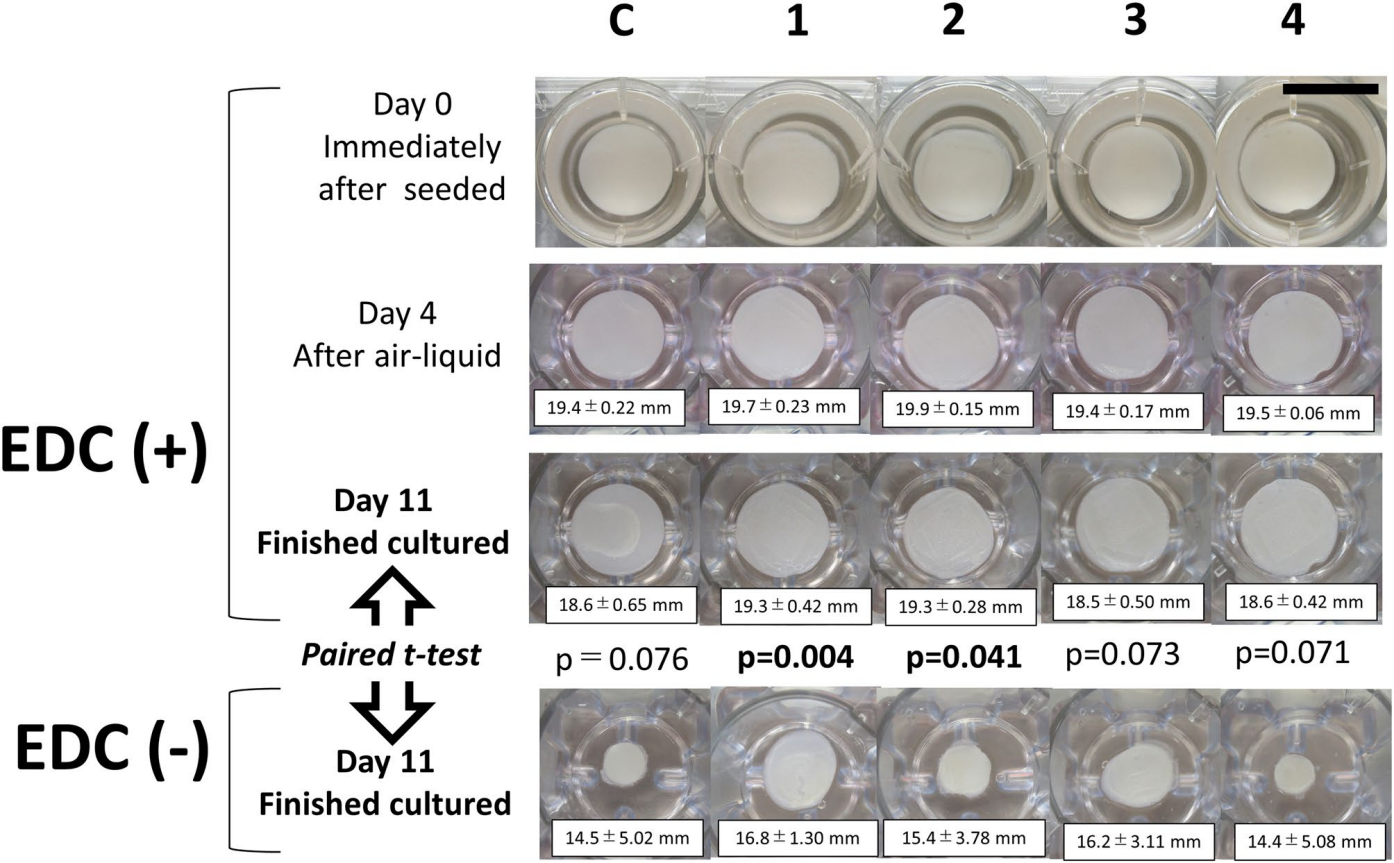
Representative macroscopic images of EVPOMEs at Day 0 (immediately after cells were seeded), Day 4 (transferred into air–liquid interface culture) and Day 11 (completion of EVPOMEs manufacturing). The initial construct size was 20 mm in diameter (Scale bar = 20 mm). Data shown are presented as mean ± SD of diameters of the EVPOMEs (n = 5). The collagen gels with EDC crosslinking showed little contraction as indicated. As p values determined by a paired t test are shown, the EVPOME dimeter was significantly reduced (contracted) after manufacturing the grid-type scaffolds (G-R and G-T) without 1% EDC crosslinking, compared with the crosslinked ones. Although the contraction of other EVPOMEs was not statistically significant, the resulting EVPOME diameters were inconsistent due to large variations.

Contraction of the EVPOMEs using collagen scaffold crosslinked with 1% EDC was rarely seen during the 11 days of manufacturing. In contrast, severe contraction was often detected in the EVPOMEs without 1% EDC crosslinking. Statistical analysis indicated the significant contraction of the EVPOMEs when the grid micropattern prototype was used (Fig. 5). In addition, there were large variations among samples without 1% EDC crosslinking; however, statistical significance was not detected. This resulted in minor variations of the final diameter of EVPOMEs with EDC treatment among the samples, producing good consistency.

### Histologic findings of EVPOME

**Figure 6:**
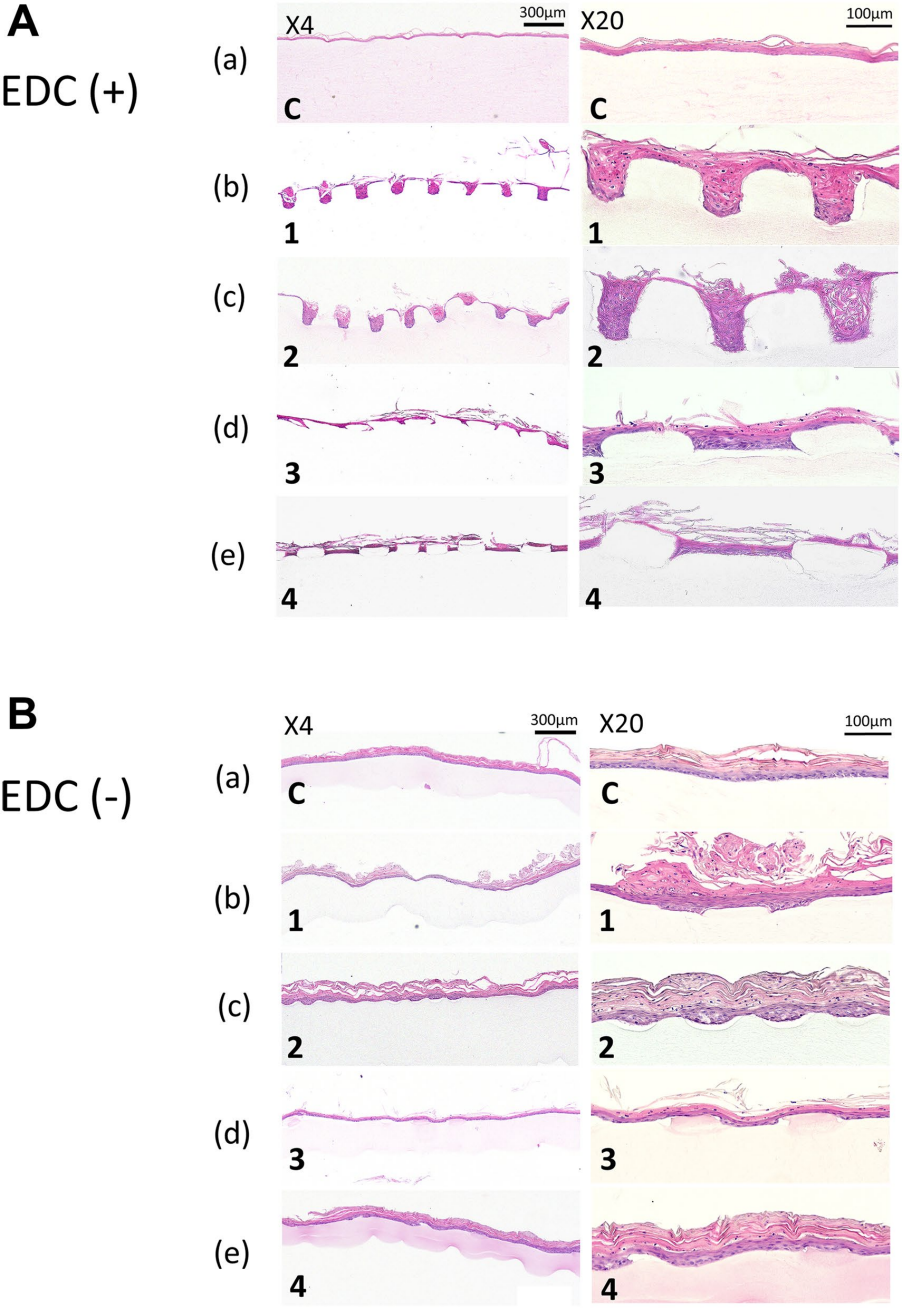
A representative histologic appearance of EVPOMEs manufactured for total 11 days, 4 days in a submerged and subsequent 7 days in an air–liquid interface condition. (n = 5): Hematoxylin and eosin staining (original magnifications, left panels 4 × , right panels 20 ×) The original microstructures fabricated on the scaffold surface cross-linked with 1% EDC were relatively well-preserved (Fig. 6A), whereas those without EDC cross-linking were hardly maintained (Fig. 6B). Additionally, epithelial thickening was observed on the scaffolds without EDC cross-linking due to the scaffold contraction. (**A**) 1% collagen scaffolds with 1% EDC crosslinking. (**a**) Non-patterned control scaffold; (**b**–**e**) indicate micropatterned collagen scaffolds originally designed by sample IDs of 1, 2, 3, and 4 shown in Fig. 1, respectively. (**B**) 1% collagen scaffolds without 1% EDC crosslinking. (**a**) Non-patterned control; (**b**–**e**) indicate micropatterned collagen scaffolds originally designed by sample IDs of 1, 2, 3, and 4 shown in Fig. 1, respectively.

First, we compared the histologic features of our EVPOMEs grown on micropatterned 1% collagen scaffolds with and without 1% EDC crosslinking (Fig. 6A,B). The dimension and aspect ratio of micropatterning were identical to the previous study design^15^. The original microstructures fabricated on the scaffold surface crosslinked with 1% EDC were relatively well-preserved, in spite of a slight decrease in the vertical dimension [Fig. 6A(b–e)], whereas those without EDC crosslinking were hardly maintained, which was characterized by an almost complete loss of their vertical dimensions [Fig. 6B(b–e)]. This finding suggested the use of 1% EDC avoided the flattening and severe deformation of the DEJ-like microstructures. In addition, compared with the scaffold without EDC crosslinking, eosinophilic collagen fibrillar structures were more evident in the scaffold crosslinked with EDC, including the portion of microstructures. This is consistent with the SEM image showing thicker fibrils with EDC crosslinking (Fig. 6A vs. B). Furthermore, a continuous and fully-differentiated epithelial layer was formed on the scaffolds with 1% EDC crosslinking [Fig. 6A(a–e)]. Remarkable epithelial thickening was also seen on all scaffolds without EDC crosslinking due to the scaffold shrinkage (contraction) during EVPOME manufacturing [Figs. 5. and 6B(a–e)]. As a result, because the configurations of the microstructures were well-maintained, the epithelial architecture developed on the EDC-crosslinked scaffold appeared to mimic an intrinsic “rete ridge” architecture. Nonetheless, because the original dimension of the micropatterns initially designed was preserved by EDC crosslinking, the undulation of the scaffold could be too large for oral keratinocytes to grow and stratify, when compared to the inherent DEJ microstructure.

**Figure 7:**
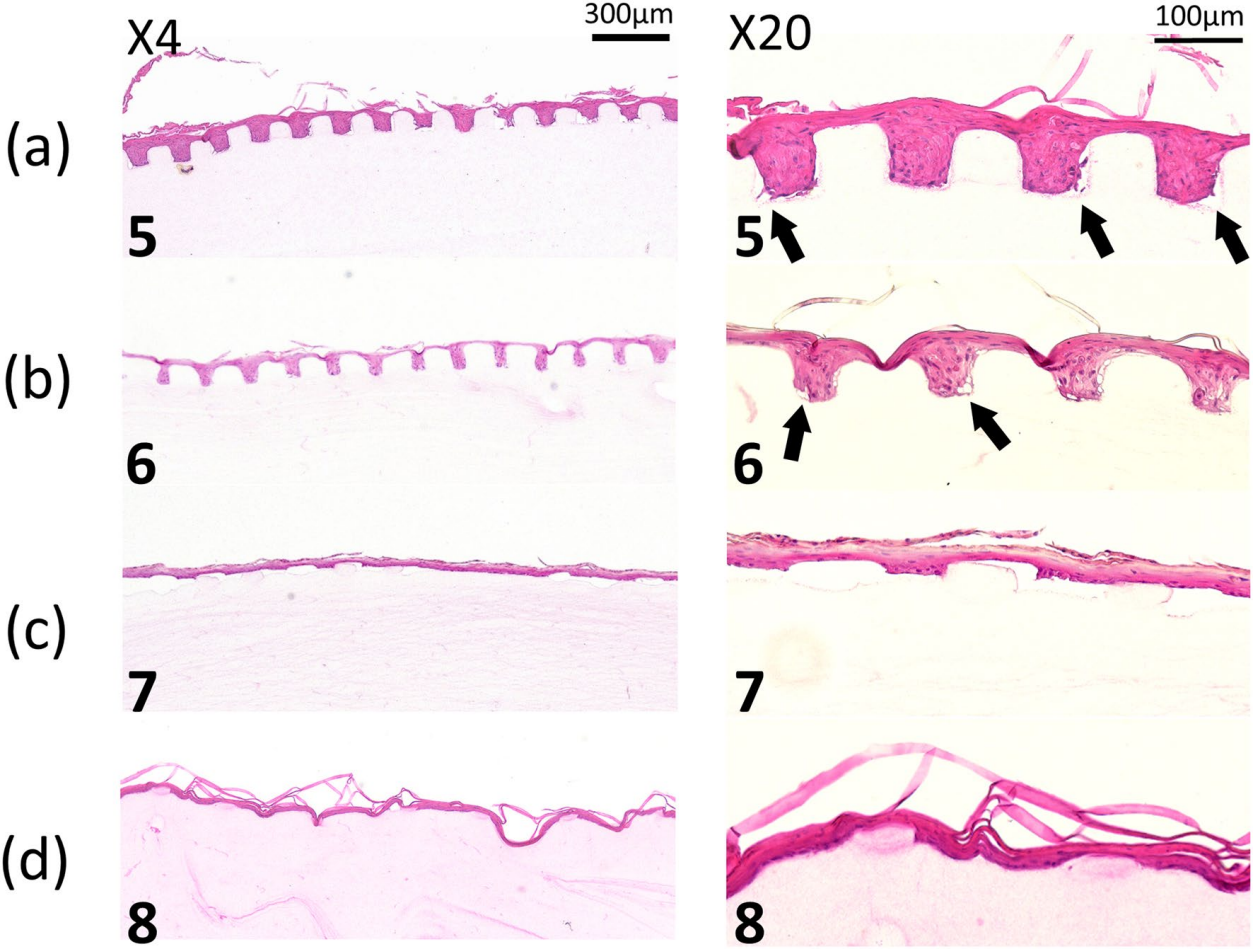
A representative histologic appearance of EVPOMEs manufactured for total 11 days, 4 days in a submerged, and subsequent 7 days in an air–liquid interface condition. (n = 5): Hematoxylin and eosin staining (original magnifications, left panels 4 × , right panels 20 ×) The grid-type micropatterns (**a**,**b**) were well-preserved and a well-stratified epithelial layer was developed. This epithelial layer showed an intrinsic rete ridge, whereas, for the pillar-type, the microstructure severely collapsed and the “rete ridge”–like structure was poorly developed, even with cross-linking with EDC. (**a**–**d**) indicate micropatterned collagen scaffolds originally designed by sample IDs of 5, 6, 7, and 8 shown in Fig. 1, respectively. Arrows indicate tiny gaps between cells and scaffold at the corner angle of micropatterns.

Therefore, we tested four prototypes with smaller and different configurations, as micropatterned scaffold crosslinked with EDC. Histologic findings showed that the grid-type micropatterns were well-preserved and a well-stratified epithelial layer developed (Fig. 7a,b). This epithelial layer showing an intrinsic rete ridge, unlike that formed when seeded on AlloDerm (data not shown), indicating the development of a more biomimetic tissue-engineered oral mucosa than that of our previous study. However, for the pillar-type, the microstructure severely collapsed and their height was almost lost, even with crosslinking with EDC (Fig. 7c,d). Apart from those findings, many tiny gaps between cells and scaffold were found at the corner angle of micropatterns.

**Figure 8:**
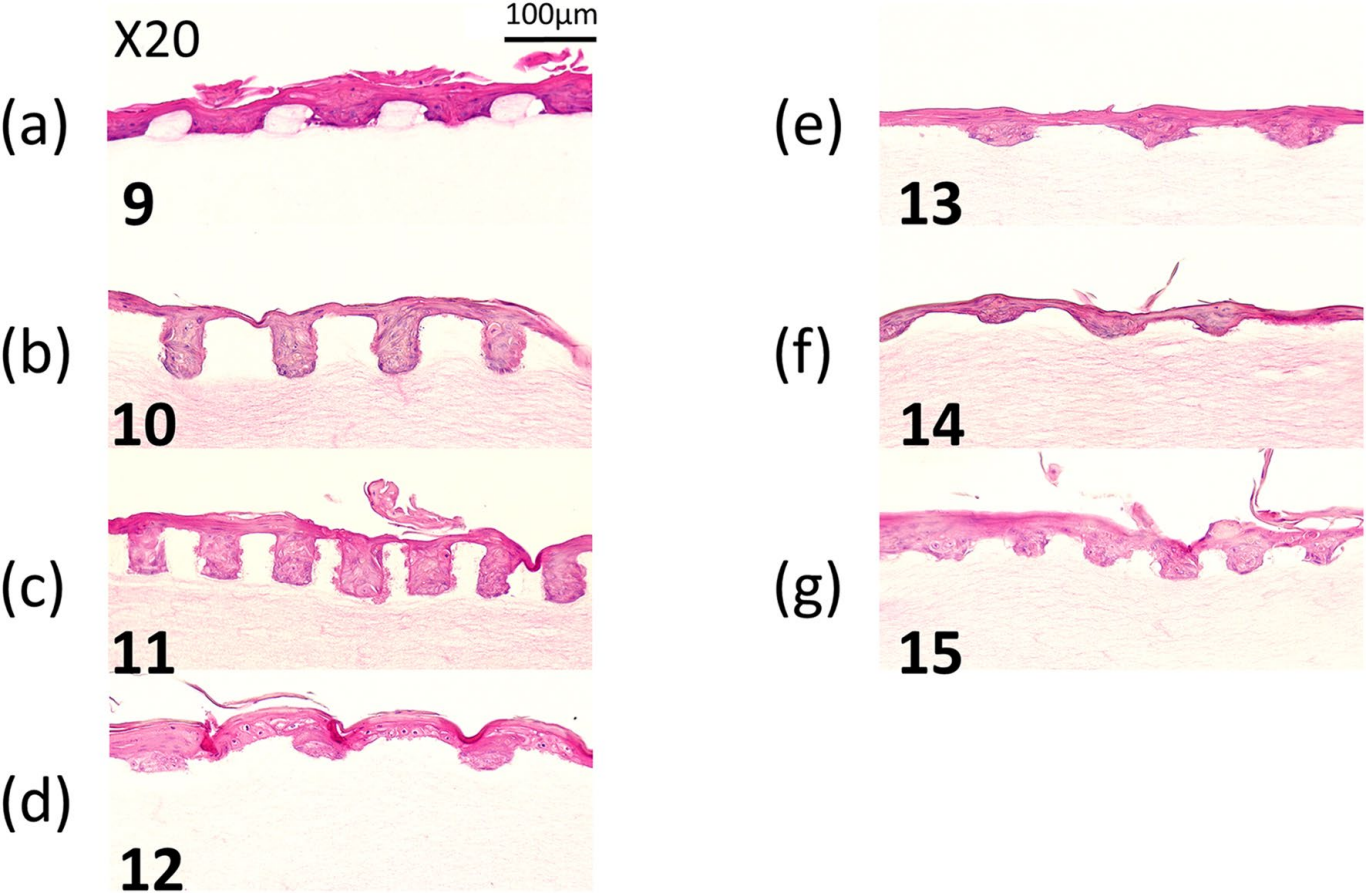
A representative histologic appearance of EVPOMEs manufactured for a total 11 of days, 4 days in a submerged, and subsequent 7 days in an air–liquid interface condition. (n = 5): Hematoxylin and eosin staining (original magnifications 20 ×) A continuous and well-stratified epithelial layer that resembles to “rete ridges” was formed. (**a**–**g**) indicate micropatterned collagen scaffolds originally designed by sample IDs of 9, 10, 11, 12, 13, 14, and 15 shown in Fig. 1, respectively.

The histologic appearances of EVPOMEs using G–R micropattern was the most stable and consistent [Fig. 6A(a,b)]. Therefore, using a 1% collagen matrix crosslinked with 1% EDC, we designed a smaller micropattern of G–R prototype, with different aspect ratios, to evaluate the conformability of our manufacturing system. A continuous and well-stratified epithelial layer that resembles to “rete ridges” was again formed on all micropatterns designed although the degree of the micropattern deformation varied depending on the aspect ratios (Fig. 8).

## Discussion

Soft lithography is one of the patterning techniques that fabricate or replicate micro/nanostructures using an “elastomeric stamp.” In this work, we have extended our previous technique, combined with MEMS process for fabricating negative molds, to engineer collagen scaffolds, applying it to different designs of DEJ-like micropattern. Several reports have shown a similar strategy to develop micropattern of intricate DEJ-like structure onto the surface of hydrogel scaffolds using photolithography as dermal substitutes for clinical application to reconstruct tissue defects^16,17^. However, their basic microarchitecture was a “grooved” pattern only, which is not as complex as the prototypes produced in this study. Compared with conventional photolithography, soft lithography methods are more high-throughput in the laboratory setting, with wider resolution, lower costs and a variety of pattern-transferring methods^18^. These advantages helped us to fabricate more complex microstructures in our system. Therefore, our system is beneficial in engineering ECM-based biomaterials for the development of a variety of the design of microstructures in negative molds for transfer onto the collagen gel, hence suggesting versatility of our system to create the micropatterning design.

Beside from soft lithography, electrospinning and 3D bioprinting, regardless of cell-free or cells-loaded, are another major micro/biofabrication technique to develop tissue-engineered soft tissue constructs^10,11^. Recent reports demonstrated that both methods have enabled the replication of the topography of the DEJ structure on hydrogel scaffolds that lack skin equivalents^19–21^. Compared with soft lithography, however, the strengths and weaknesses of these approaches are not clear due to growing advancements of these nascent micro/biofabrication technology for dermal substitutes^12,22,23^. Further comparison and evaluation among these state-of-art technologies are needed to consider how different techniques will affect the microenvironmental cues for directing cellular behaviors, eventually leading to applications for engineered soft tissue constructs^24^.

For tissue engineering applications, covalent intermolecular crosslinks between collagen molecules using appropriate biocompatible molecules are essential to develop stable biomaterials with a high degree of mechanical integrity^25^. EDC used in this study has also been applied to marine derived collagen/gelatin^26^. One of the direct effects was to inhibit gel contraction after oral keratinocytes were seeded, which allowed the omission of 1% chondroitin sulfate into collagen that was added in our previous study. Also, consistent with other studies, the enhancement of stiffness provided the collagen matrix with more durability and manageability of the scaffold that enabled suture while avoiding rupture of the scaffold^27,28^. This property, which can be contributed by thicker collagen fibrils as compared with those without cross-linking, is significant for technicians in cell-processing centers and surgeons. Although chemically crosslinked hydrogels are apt to be less biocompatible than physically crosslinked ones due to the potential cytotoxicity of the residual chemicals and reagents, EDC has been utilized in many soft tissue-engineered products, including CollaMend and Sharklet. These facts indicate insignificance of EDC cytotoxicity^17,29–31^. However, the stability of collagen gel treated with EDC decreases the degradation rate in vivo^32,33^. An in vivo evaluation of our EVPOMEs is required for future clinical applications.

This study confirmed that the Tilapia collagen scaffold has a characteristic of an ideal gel by the rheological test, consistent with the animal-derived ones, suggesting the scaffolds fabricated in this study are visco-elastic, shown to be biomimetic^34–36^. On the other hand, this visco-elastic property makes it difficult to accurately evaluate the configuration of micro-scale fine structures, such as the DEJ-like undulations, because the fixatives used for SEM and histological observation cause sample contraction, resulting in artefacts. To obviate deformation of the specimens, such as buckling of microstructures, it is necessary to develop a live 3D imaging technology to measure soft tissue constructs non-invasively^37,38^.

A major achievement of this study was the successful development of biomimetic oral mucosa equivalents having a rete ridge–like epithelial structure when the cells were seeded onto the grid-type microstructures showing minimal deformation. Nevertheless, similar to our previous histologic evaluations, the microscopic findings of EVPOMEs again showed incomplete shape fidelity of the scaffold surface micropatterns, exhibiting partial failure to replicate the DEJ-like microarchitecture originally designed in the negative molds while it was obvious that the grid-type microstructures were more stable with minimal reduction of the vertical dimension than the pillar-types. Although this difference could be due to mechanical structural issue and/or different eosinophilic fibril distribution between the two prototype designs, this defect causes the failure of the comprehensive evaluation to investigate the effect of micropatterning-based cues over oral keratinocytes proliferation, differentiation, and stem cell niche, an area that needs to be explored and studied for regenerative medicine^39^. Therefore, it is necessary to address the incomplete shape fidelity of microstructures on collagen scaffold to advance our technology. Although EDC crosslinking improved the mechanical properties of the scaffold, the portion of the micropatterns did not seem to have sufficient physical property to tolerate the mechanical forces during epithelial formation^39,40^. To manufacture a stiffer collagen scaffold, another approach, such as producing basement membrane-like structure or use of supramolecular cross-linkers, may be required to tolerate the mechanical force generated by cells, producing a stable microenvironmental cue^41^.

The serrated interface between basal cells and the collagen scaffolds was not present in this study. Mechanical properties of hydrogel are critical for the stability of the scaffold in culture and should have an impact on cellular mechano-transduction regulated by traction force or F-actin distribution of keratinocytes^42–44^. Therefore, the histologic finding may depend on the elastic module of the scaffold, suggesting that the mechanical property must also be engineered when the micropatterning is designed using ECM-based biomaterials.

Histologic evaluation reveals there were small cleavages present at the corners of the indentations between cells and collagen surface. The fact that cells did not attach to the scaffold implies that there is a need to eliminate the right angle planes on the scaffold. Since most physiological occurring structures, such as DEJ and microvilli, are inherently curved, the topography and curvature along villus-like microstructures have a significant effect on the cellular morphology of small intestinal epithelial cells in physiological events^6^. Thus, engineering 3D curvature (locally) on an undulating microstructure of the scaffold may be necessary to develop better biomimetic oral mucosa equivalents.

Due to using a marine byproduct for the production of collagen, marine origin materials, basically a waste product in the fishery industry, emerged as a sustainable resource^45^. Therefore, marine collagen, such as Nile Tilapia, is one of the major collagen sources, which can be alternative to mammalian-derived collagen. Marine collagen is a safer and more attractive biomaterial for processing using advanced fabrication techniques, confirming the growing relevance of marine biomaterials in tissue engineering and regenerative medicine^46–50^. Although further research is needed, the skin of Nile Tilapia had a therapeutic effect on burn wounds in human clinical trials^51^. Therefore, the collagen scaffold manufactured in this study could facilitate wound healing after transplantation onto wounds in the oral cavity. Applications of marine collagen as a biomimicking material, not only for hard tissues but also soft tissues including hemostats, has great potential in regenerative medicine^52,53^.

In summary, our manufacturing system is valuable for fabricating a negative mold to replicate a variety of the biomimetic microstructures, such as DEJ on collagen scaffolds, implying competency of this technique to engineer scaffolds with a micropattern composed of a variety of hydrogels, such as the native topography of DEJ. Together, with easy-handling and durability against suturing, the physical properties of the scaffold gained by using 1% EDC crosslinking make the 1% tilapia scale collagen a suitable biomaterial for future clinical applications in regenerative medicine, especially in epithelial tissue defects. Although in vivo studies of the micropatterned collagen scaffold are necessary to examine its immunogenicity and biodegradability, and effects on epithelial regeneration, this scaffold can be applied as acellular substitutes for intraoral and extraoral epithelial tissue reconstruction and regeneration. Furthermore, this construct of engineered oral mucosa with rete ridges could be utilized as an in vitro system to study oral keratinocyte stem cells, a test for pharmaceutical evaluation in full-thickness wound repair, when more stable properties are provided onto the portion of microstructure.

## Methods

### Ethical approval

The use of human oral mucosa keratinocytes and the procurement procedure was approved by the Internal Review Board of the Niigata University Hospital. Number: 2015–5018. All methods were carried out in accordance with relevant guidelines and regulations.

### Design of micropatterns and fabrication of microstructured negative molds

In order to mimic the connective tissue papilla of oral mucosa, we previously designed two grid- and pillar-micropattern prototypes with rectangular or truncated configurations, respectively, resulting in four negative molds as micropatterns, and their dimensions on the scaffold designed were approximately 200 μm in height, 200 μm in thickness, and 100 μm in channel width^15^. To test the conformability of our manufacturing system, 15 different dimensions and aspect ratios smaller than those of previous study, including the same four micropatterns (i.e., ratio of topography height to topography thickness), were manufactured. The dimensions were simply changed with 50% reduction of the height and channel width of a grid micropattern and 50% reduction of the height and thickness of a pillar micropattern. In addition, all combinations of height, thickness and channel width of 100 μm and 50 μm were applied to micropattern because of the structural stability of G–R prototype. Negative molds designed in this study were fabricated as described previously^15^. Briefly, an initial silicon (Si) mold for grid patterns was prepared via anisotropic deep-reactive ion etching using a photoresist mask, and then Si isotropic wet etching was performed using an acid mixture to form the truncated structure. Subsequently, the polydimethylsiloxane (PDMS, SILPOT 184, Dow Corning Toray, Tokyo, Japan) was casted against the Si substrate with grid patterns to form a soft lithography mold. Si substrates with through-hole patterns, which were mainly fabricated via anisotropic deep-reactive ion etching, were directly used as negative molds for pillar patterns.

### Fabrication and measurement of physical property of collagen scaffolds, and macroscopic test of handling property

A total of 16 scaffolds comprising four different micropattern prototypes with different dimensions, including a flat surface control, comprising 1% type I tilapia scale atelocollagen matrix with or without EDC crosslinking, were prepared^54^. Cell campus (100% freeze-dried collagen: FD-08G, Taki Chemical Co., Ltd., Hyogo, Japan) was dissolved in HCl (pH 3.0) at 1.1 wt%. The collagen solution was mixed with Dulbecco’s phosphate buffered saline (D-PBS, KAC Co., Ltd., Kyoto, Japan) at 4 °C. After pouring each collagen matrix solution into the PDMS or Si, molds were inverted and immersed into each collagen solution, both molds were placed into an incubator (25 °C) to induce fibrogenesis. Subsequently, half of the collagen gels were chemically crosslinked by 1-ethyl-3-(3-dimethylaminopropyl) carbodiimide hydrochloride (EDC) (Tokyo Chemical Industry, Tokyo, Japan) treatment. The EDC was dissolved in 99.5% ethanol (Kishida Chemical) at 1.0%w/v. EDC crosslinking was performed by immersing the micropatterned collagen gels in a solution containing 100 mg of EDC per 7.8 mg of type I collagen at room temperature for 24 h. After crosslinking, the scaffolds were placed individually in a container and washed in D-PBS by rotational stirring at room temperature for 24 h. Then, they were all γ-irradiated for sterilization. For simplifying scaffold fabrication, the surrounding collagen matrix surface of the 14-mm square-shaped microstructure was planarized^15^.

Five pieces of 1% collagen gels with or without 1% EDC crosslinking, without microstructures having a diameter of 20 mm and a thickness of 2 mm, were used for measuring storage modulus and loss modulus. They were determined by means of a rheometer (HAAKE MARS III, Thermo Fisher Scientific Inc., Germany) with a compression speed of 0.2 mm/s, at 37 °C. In addition, Young’s modulus was determined using a compact table-top universal tester (EZ-LX, Shimazu Corporation, Kyoto, Japan) with a compression speed of 0.15 mm/s, at 37 °C. Subsequently, the handling property of the collagen gels without or with EDC cross-linking was tested. After only the epithelial layer, 20 mm in diameter, was removed from Bio-SKiN, a medical training artificial skin (Regina Fashion Supply Co. Ltd., Saitama, Japan), the gels were placed on the “wounded” surface. Using 4–0 braided silk (Nesco, Tokyo, Japan) and surgical instruments, the collagen gel (transplant) was sutured with the surrounding Bio-SKiN, supposed to be oral mucosa, and photographed.

### Observation of negative molds and 1% collagen gels crosslinked with 1% EDC

The top views of the negative molds for grid patterns (PDMS) and pillar patterns (Si) were observed either optical microscopy (VH-S30, KEYENCE, Osaka, Japan) or confocal microscopy (K-9510, KEYENCE), respectively (n = 3). In addition, we observed the microstructures of the 1% tilapia scale type I collagen matrices crosslinked with 1% EDC using scanning electron microscopy (SEM) as described previously^15^.

### Procurement of oral mucosa samples

The protocol for obtaining human oral mucosa samples was approved by the Niigata University Hospital Internal Review Board (approval # 2015–5018). Patients receiving minor dentoalveolar surgery at an oral and maxillofacial surgery outpatient clinic at Niigata University Hospital were provided with sufficient information regarding this study, and all participating individuals signed an informed consent form. An oral mucosa tissue sample, approximately 5 mm^2^ in size, was trimmed off from the elevated mucoperisoteal flap.

### Cell culture of primary oral mucosa keratinocytes

Primary oral mucosa keratinocytes were serially cultured as previously described^15^. Briefly, the tissue sample was soaked in a 0.025% trypsin/EDTA solution (Thermo Fisher Scientific, Waltham, MA, USA) containing 1.5% Antibiotic–Antimycotic (Thermo Fisher Scientific), overnight at room temperature. Oral mucosa keratinocytes were scraped off from the underlying connective tissue using a scalpel in a 0.0125% defined trypsin inhibitor (Thermo Fisher Scientific), resuspended in EpiLife supplemented with EpiLife Defined Growth Supplements (Thermo Fisher Scientific), referred to as “complete medium,” and plated at a density of 4.0–5.0 × 10^4^ cells/cm^2^. After reaching a confluence of 70%–80%, the cells were re-plated at a density of 0.7–1.0 × 10^4^ cells/cm^2^, fed with the complete medium supplemented with gentamicin (5.0 μg/mL), and amphotericin B (0.375 μg/mL; Thermo Fisher Scientific). Oral mucosa keratinocytes from passages 3 to 5 were used in the study.

### Manufacturing of tissue-engineered oral mucosa equivalents (EVPOMEs)

The EVPOMEs were manufactured by seeding oral mucosa keratinocytes obtained from five individuals onto microstructured tilapia scale collagen scaffolds having four microstructures with various aspect ratios (with one flat surface as a control), with or without 1% EDC crosslinking. AlloDerm (Allergan, Madison, NJ, USA) was used as a positive control^15^. According to our human clinical application protocol, after presoaking the scaffolds in type IV collagen (5 μg/cm^2^, derived from the human placenta, Sigma-Aldrich) in D-PBS (Wako chemical, Osaka, Japan) overnight at 4 °C in a 12-well plate, oral mucosa keratinocytes were seeded onto all scaffolds at a cell density of 1.5 × 10^5^ cells/cm^2^^55^. The composites were cultured in complete medium supplemented with 1.2 mM Ca^2+^ for 4 days in a submerged condition, and then raised to an air–liquid interface with the same culture medium for another 7 days^15^.

### Macroscopic analysis of EVPOMEs and statistical analysis

During manufacturing the EVPOMEs, their diameters were measured every day, as previously stated using Image J (National Institutes of Health, Bethesda, MD, USA, http://imagej.nih.gov/ij/)^15^. A paired *t*-test was performed to analyze the contraction of the day 11 EVPOMEs (diameter) fabricated on scaffolds without crosslinking, as compared with those treated with 1% EDC using the Excel software (n = 5). A *p* value < 0.05 was considered significantly different.

### Histologic examination of EVPOMEs

A total of 31 EVPOMEs were fixed with 4% paraformaldehyde in 100 mM D-PBS and embedded in paraffin. The paraffin-embedded samples were deparaffinized, rehydrated, cut into 5-µm thick sections, and stained with hematoxylin and eosin for histologic examination.

## Data Availability

The data presented in this study can be provided by the corresponding author upon reasonable request due to pending patent application.
